# Gamma-glutamyl-transpeptidase to platelet ratio is not superior to APRI,FIB-4 and RPR for diagnosing liver fibrosis in CHB patients in China

**DOI:** 10.1038/s41598-017-09234-w

**Published:** 2017-08-17

**Authors:** Rui Huang, Guiyang Wang, Chen Tian, Yong Liu, Bei Jia, Jian Wang, Yue Yang, Yang Li, Zhenhua Sun, Xiaomin Yan, Juan Xia, Yali Xiong, Peixin Song, Zhaoping Zhang, Weimao Ding, Chao Wu

**Affiliations:** 1 Department of Infectious Diseases, Nanjing Drum Tower Hospital, Nanjing University Medical School, Nanjing, Jiangsu China; 2 Department of Laboratory Medicine, Nanjing Drum Tower Hospital, Nanjing University Medical School, Nanjing, Jiangsu China; 3 Department of Hepatology, Huai’an No. 4 People’s Hospital, Huai’an, Jiangsu China

## Abstract

The gamma-glutamyl transpeptidase to platelet ratio (GPR) is a novel index to estimate liver fibrosis in chronic hepatitis B (CHB). Few studies compared diagnostic accuracy of GPR with other non-invasive fibrosis tests based on blood parameters. We analyzed diagnostic values of GPR for detecting liver fibrosis and compared diagnostic performances of GPR with APRI (aspartate aminotransferase-to-platelet ratio index), FIB-4 (fibrosis index based on the four factors), NLR (neutrophil-to-lymphocyte ratio), AAR (aspartate aminotransferase/alanine aminotransferase ratio) and RPR (red cell distribution width-to-platelet ratio) in HBeAg positive CHB and HBeAg negative CHB. We found AUROCs of GPR in predicting significant liver fibrosis, advanced liver fibrosis and liver cirrhosis were 0.732 (95% CI 0.663 to 0.801), 0.788 (95% CI 0.729 to 0.847) and 0.753 (95% CI 0.692 to 0.814), respectively. Further comparisons showed the diagnostic performance of GPR was not significantly different with APRI, FIB-4 and RPR in identifying significant fibrosis, advanced fibrosis and cirrhosis, but it was significantly superior to AAR and NLR in both HBeAg positive CHB and HBeAg negative CHB. In conclusion, GPR does not show advantages than APRI, FIB-4 and RPR in identifying significant liver fibrosis, advanced liver fibrosis and liver cirrhosis in both HBeAg positive CHB and HBeAg negative CHB in China.

## Introduction

Chronic hepatitis B virus (HBV) infection is a major public health problem. Patients with chronic HBV infection have a high risk of progressive liver fibrosis, cirrhosis and even hepatocellular carcinoma (HCC)^1, 2^. Therefore, the early diagnosis of liver fibrosis or cirrhosis and treating immediately plays an important role in the control of disease progression and may decrease the morbidity and mortality of end stage liver diseases related to HBV infection^3^. Fibrosis staging is an essential step in the clinical assessment of patients with HBV infection to identify those who require treatment. Liver biopsy (LB) is considered to be the gold standard method to stage the degrees of fibrosis^3^. However, it is restricted the widespread utilization by its invasiveness, patient discomfort, risk of complications, contraindications and sampling error^3–6^. Furthermore, it does not allow the dynamic observation of liver fibrosis by LB. Recently, transient elastography (TE) was introduced as a noninvasive, highly reproducible technique for evaluating and staging liver fibrosis, which relatively accurately assess the degree of liver fibrosis and may reduce the need for LB^7, 8^. However, despite the clinical usefulness of TE, several confounding factors such as necroinflammatory activity, total bilirubin, cholestasis, as well as the skill of the operators may diminish the accuracy of TE^9–11^. Moreover, the high cost of the equipment, the need for preventive and corrective maintenance and the lack of extensively validated cut-off values for specific stages of fibrosis may also limit the clinical use of TE^12^.

Several non-invasive fibrosis tests (NITs) based on blood or serum parameters such as aspartate transaminase (AST)-to-platelet ratio index (APRI) and the fibrosis index based on the four factors (FIB-4) which have the advantage of comprising only two or three inexpensive laboratory tests are recommend as non-invasive tools to detect significant fibrosis in resource-limited settings in World Health Organization (WHO) guidelines^12^. However, APRI and FIB-4 can only identify HBV-related fibrosis with a moderate sensitivity and accuracy^13, 14^. Recently, the gamma-glutamyl transpeptidase (GGT) to platelet ratio (GPR) had been developed to be a novel and more accurate routine laboratory marker than classical biomarkers APRI and FIB-4 to estimate liver fibrosis in patients with chronic hepatitis B (CHB) in West Africa cohorts (Gambia cohort and Senegal cohort), but it was not superior to APRI and FIB-4 in a French cohort^15^. The GPR also did not show any advantage in a Brazilian cohort and a Chinese cohort^16, 17^. Furthermore, several novel models based on blood or serum parameters such as AST/alanine aminotransferase (ALT) ratio (AAR), neutrophil-to-lymphocyte ratio (NLR) and red cell distribution width (RDW)-to-platelet ratio (RPR) have been proposed to predict significant fibrosis and cirrhosis over the past decade in CHB patients with relatively high accuracy^18^. However, few studies compared the difference of diagnostic accuracy of GPR with these NITs.

In the present study, we analyzed the diagnostic values of GPR for significant liver fibrosis (≥F2), advanced liver fibrosis (≥F3) and liver cirrhosis (F4) in CHB patients in China and compared the diagnostic performances of GPR with other NITs including APRI, FIB-4, NLR, AAR and RPR. Furthermore, we compared the diagnostic accuracy of GPR with other NITs in both hepatitis B e antigen (HBeAg) positive CHB and HBeAg negative CHB in the present study.

## Results

### Study population

Three hundred and thirty-two patients with CHB were enrolled. Supplementary Figure S1 summarizes the flow diagram of the study population. Of these, 27 patients were excluded based on exclusion criteria, and 49 patients were excluded because of inappropriate biopsy samples or insufficient data. Two hundred and fifty-six CHB patients who met the eligibility criteria were included as study subjects for the following analysis. The demographic and clinical characteristics of the subjects are shown in Table 1. The median (and IQR) age of the CHB patients was 38.0 (29.0–46.0) and 79.7% of the patients was male. 142/256 (55.5%) CHB patients were positive for HBeAg and the median (and IQR) HBV DNA levels was 5.3 (3.3, 6.8) log10 copies/ml. The median (and IQR) ALT and AST levels were 42.0 (28.0, 79.8) IU/L and 36.0 (26.0, 58.5) IU/L, respectively. The distribution of fibrosis stages was: F0 (13.3%), F1 (16.0%), F2 (12.5%), F3 (25.8%) and F4 (32.4%).

**Table 1:** Baseline characteristics of the study population.

Characteristic	CHB
	(n = 256)
Median age (years) (IQR)	38.0 (29.0, 46.0)
Male (%)	204 (79.7)
Median ALT (IU/L) (IQR)	42.0 (28.0, 79.8)
Median AST (IU/L) (IQR)	36.0 (26.0, 58.5)
Median GGT (IU/L) (IQR)	38.0 (23.0, 78.8)
Median Neutrophils (×10^9^/L) (IQR)	2.9 (2.2, 3.6)
Median Lymphocytes (×10^9^/L) (IQR)	1.7 (1.4, 2.1)
Median Hb (g/L) (IQR)	145.0 (132.3, 154.0)
Median PLT (×10^9^/L) (IQR)	148.5 (110.5, 190.5)
Median RDW (%) (IQR)	12.0 (11.5, 12.6)
Tbil (μmol/L) (IQR)	15.1 (11.6, 21.5)
HBeAg positive (%)	142 (55.5)
Median size of liver biopsy (cm) (IQR)	1.3 (1.0, 1.5)
Fibrosis stages	
F0 (%)	34 (13.3)
F1 (%)	41 (16.0)
F2 (%)	32 (12.5)
F3 (%)	66 (25.8)
F4 (%)	83 (32.4)
Median HBV DNA level (log10 copies/mL) (IQR)	5.3 (3.3, 6.8)

ALT, alanine aminotransferase; AST, aspartate aminotransferase; CHB, chronic hepatitis B; GGT, gamma-glutamyl transferase; Hb, hemoglobin; IQR, interquartile range; PLT, platelets; RDW, red cell distribution width; Tbil, total bilirubin.

### Comparisons of different NITs according to the fibrosis stages in CHB patients

**Figure 1:**
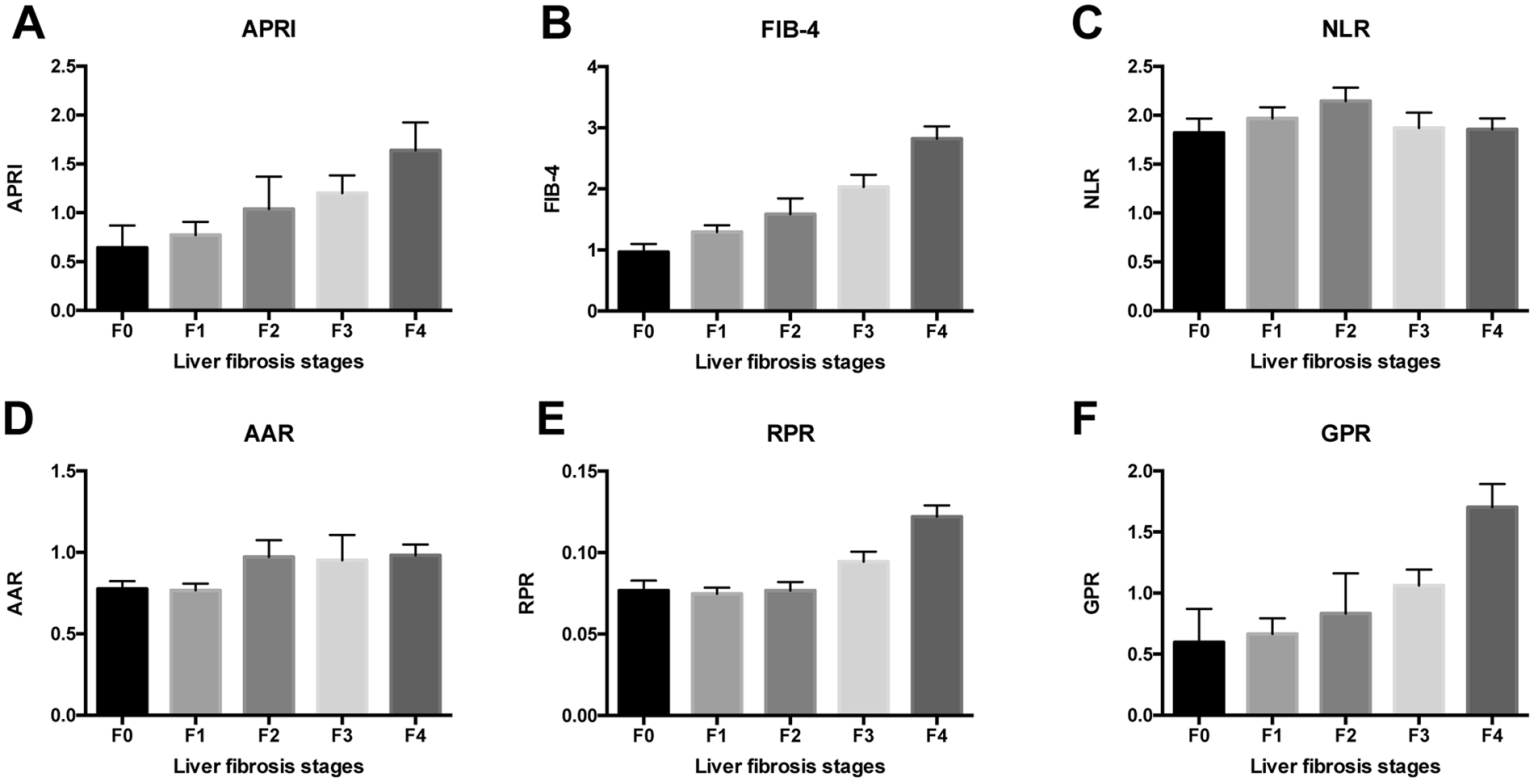
Comparisons of different NITs according to the fibrosis stages in CHB patients.

Comparisons of different NITs according to the fibrosis stages in CHB patients are presented in Fig. 1. The APRI, FIB-4, RPR and GPR showed an increasing trend with fibrosis stages in CHB patients. The correlations of six NITs with liver fibrosis stages were analyzed by the Spearman’s rank correlation coefficient analysis. The GPR (r = 0.511, P < 0.001) and established non-invasive markers including APRI (r = 0.404, P < 0.001), FIB-4 (r = 0.503, P < 0.001), AAR (r = 0.167, P = 0.007) and RPR (r = 0.421, P < 0.001) were positively correlated with liver fibrosis stages. However, the NLR (r = −0.106, P = 0.091) was not correlated with liver fibrosis stages (Supplementary Figure S2).

### Comparisons of diagnostic performance between GPR and other established NITs in the entire CHB population

**Figure 2:**
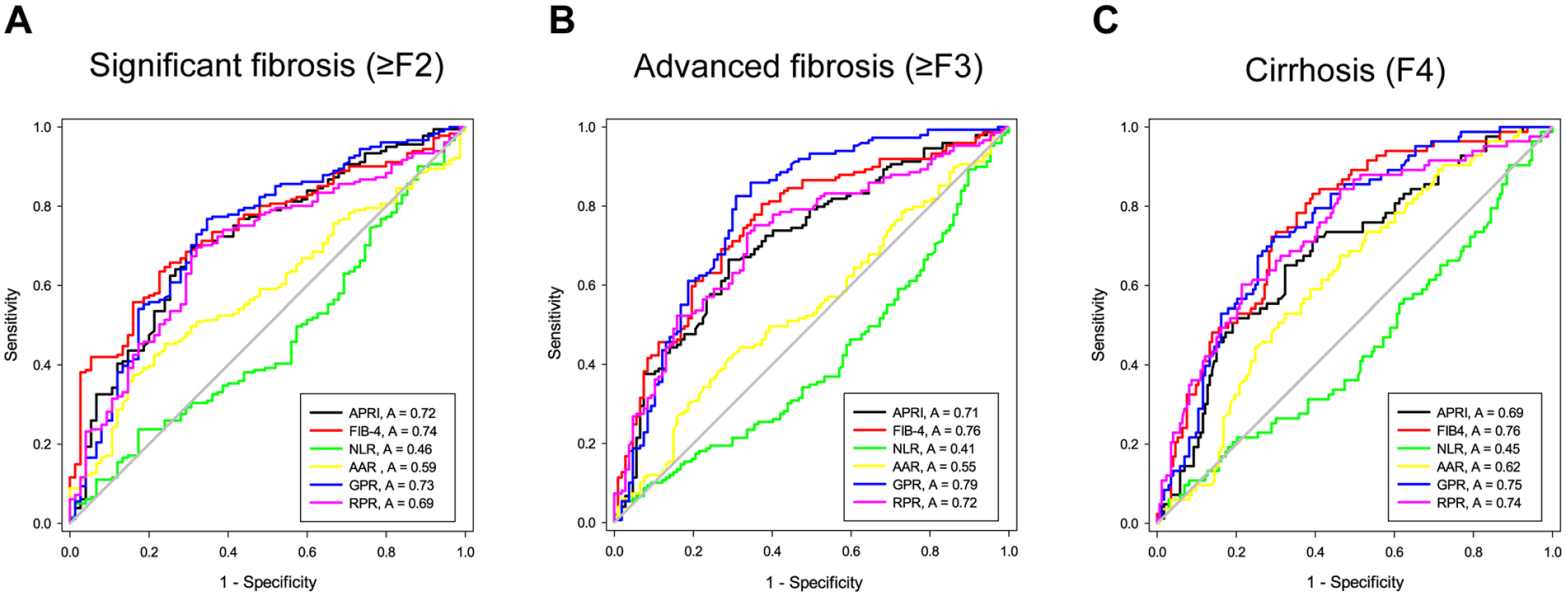
Receiver operating characteristic (ROC) curves of APRI, FIB-4, NLR, AAR, RPR and GPR in the prediction of significant liver fibrosis (**A**), advanced liver fibrosis (**B**) and liver cirrhosis (**C**) for the entire CHB patients.

The ROC curves of six models for predicting significant liver fibrosis (≥F2), advanced liver fibrosis (≥F3) and liver cirrhosis (F4) in the entire CHB population are shown in Fig. 2. The diagnostic performances of different NITs are presented in Table 2.

**Table 2:** Diagnostic accuracy of different indexes for the prediction of liver fibrosis in CHB patients.

	Optimized cutoff	Sensitivity (%)	Specificity (%)	AUROC (95% CI)	LR+	LR−	P value	P value of ROC contrast test*
**F0-F1 vs**. **F2-F4**								
APRI	0.561	68.51	70.67	0.719 (0.650, 0.787)	2.336	0.446	<0.001	0.640
	0.5*	74.59	58.67	—	1.804	0.433	<0.001	—
	1.5*	25.41	94.67	—	4.765	0.788	<0.001	—
FIB-4	1.411	63.54	77.33	0.741 (0.679, 0.806)	2.803	0.472	<0.001	0.777
	1.45*	61.33	77.33	—	2.706	0.500	<0.001	—
	3.25*	20.99	97.33	—	7.873	0.812	<0.001	—
NLR	2.301	23.76	82.67	0.465 (0.388, 0.541)	1.371	0.922	0.371	<0.001
AAR	0.952	37.57	84.00	0.586 (0.513, 0.658)	2.348	0.743	0.031	0.013
RPR	0.075	69.61	68.00	0.691(0.621, 0.760)	2.175	0.447	<0.001	<0.001
GPR	0.341	76.80	65.33	0.732 (0.663, 0.801)	2.215	0.355	<0.001	—
**F0-F2 vs**. **F3-F4**								
APRI	0.657	66.44	71.03	0.713 (0.649, 0.777)	2.293	0.473	<0.001	0.008
FIB-4	1.098	80.54	62.62	0.757 (0.697, 0.817)	2.155	0.311	<0.001	0.339
NLR	3.611	5.37	98.13	0.409 (0.338, 0.479)	2.871	0.964	0.013	<0.001
AAR	0.888	41.61	71.03	0.545 (0.474, 0.617)	1.436	0.822	0.216	<0.001
RPR	0.075	75.17	64.49	0.721 (0.658, 0.787)	2.117	0.385	<0.001	0.053
GPR	0.413	82.55	69.16	0.788 (0.729, 0.847)	2.677	0.252	<0.001	—
**F0-F3 vs**. **F4**								
APRI	0.780	65.06	67.63	0.686 (0.616, 0.755)	2.010	0.517	<0.001	0.020
	1.0*	51.81	78.61	—	2.422	0.613	<0.001	—
	2.0*	19.28	90.17	—	1.962	0.895	0.035	
FIB-4	1.341	83.13	60.69	0.762 (0.702, 0.823)	2.115	0.278	<0.001	0.769
NLR	3.611	6.02	97.11	0.446 (0.370, 0.523)	2.084	0.968	0.165	<0.001
AAR	0.767	67.47	53.76	0.622 (0.552, 0.693)	1.459	0.605	0.002	0.012
RPR	0.096	60.24	78.61	0.738 (0.671, 0.804)	2.816	0.506	<0.001	0.655
GPR	0.637	72.29	70.52	0.753 (0.692, 0.814)	2.452	0.393	<0.001	—

*Compared with GPR.

AUROC, area under the receiver operating characteristic curve; CI, confidence interval; LR−, negative likelihood ratio; LR+, positive likelihood ratio; AAR, aspartate aminotransferase/alanine aminotransferase ratio; APRI, aspartate aminotransferase-to-platelet ratio index; FIB-4, fibrosis index based on the four factors; GPR, gamma-glutamyl transpeptidase to platelet ratio; NLR, neutrophil-to-lymphocyte ratio; RPR, red cell distribution width-to-platelet ratio.* threshold recommended by the WHO^12^.

The AUROCs of GPR in predicting significant fibrosis, advanced fibrosis and liver cirrhosis were 0.732 (95% CI 0.663 to 0.801), 0.788 (95% CI 0.729 to 0.847) and 0.753 (95% CI 0.692 to 0.814), respectively. The optimal cut-off values of GPR for predicting significant fibrosis, advanced fibrosis and liver cirrhosis were 0.341, 0.413 and 0.637.

For predicting significant fibrosis, AUROC of GPR was significantly higher than of NLR (0.465, 95% CI 0.388 to 0.541, P < 0.001), AAR (0.586, 95% CI 0.513 to 0.658, P = 0.013) and RPR (0.691, 95% CI 0.621 to 0.760, P < 0.001), but was comparable with APRI (0.719, 95% CI 0.650 to 0.787, P = 0.640) and FIB-4 (0.741, 95% CI 0.679 to 0.806, P = 0.777).

For predicting advanced fibrosis, AUROC of GPR was higher than that of APRI (0.713, 95% CI 0.649 to 0.777, P = 0.008), NLR (0.409, 95% CI 0.338 to 0.479, P < 0.001) and AAR (0.545, 95% CI 0.474 to 0.617, P = 0.013), but was comparable with that of FIB-4 (0.757, 95% CI 0.697 to 0.817, P = 0.339) and RPR (0.721, 95% CI 0.658 to 0.787, P = 0.053).

For predicting cirrhosis, AUROC of GPR was higher than that of APRI (0.686, 95% CI 0.616 to 0.755, P = 0.020), NLR (0.446, 95% CI 0.370 to 0.523, P < 0.001) and AAR (0.622, 95% CI 0.552 to 0.693, P = 0.012), but was similar with FIB-4 (0.762, 95% CI 0.702 to 0.823, P = 0.769) and RPR (0.738, 95% CI 0.671 to 0.804, P = 0.655).

We also analyzed the diagnostic performances of APRI and FIB-4 according to the thresholds recommended by the WHO^12^ (Table 2). The sensitivities and specificities were consistent with the WHO CHB guidelines. The APRI and FIB-4 could detect significant fibrosis and cirrhosis with only moderate sensitivities and specificities by the lower thresholds. For APRI and FIB-4, the higher threshold yielded a sensitivity of merely 20%, although the specificity was high at nearly 100%. GPR was not significantly better than the WHO recommended non-invasive tests for detection of fibrosis.

### Comparisons of diagnostic performance between GPR and other established NITs in HBeAg positive CHB and HBeAg negative CHB

The baseline characteristics between HBeAg positive CHB and HBeAg negative CHB were presented in Supplementary Table S1. The median ages of HBeAg negative CHB (43.5, IQR 36.0 to 48.0) were significantly higher than that of HBeAg positive CHB (34.0, IQR 27.0 to 42.0, P < 0.001). The ALT (P < 0.001) and AST (P < 0.001) levels in the HBeAg positive CHB were significantly higher than that of HBeAg negative CHB. The median levels of HBV DNA were also higher in the HBeAg positive CHB (6.6, IQR 5.4 to 7.4) compared to HBeAg negative CHB (3.3, IQR 3.0 to 4.8, P < 0.001). However, the distributions of liver fibrosis stages were similar between these two groups (P = 0.508).

**Figure 3:**
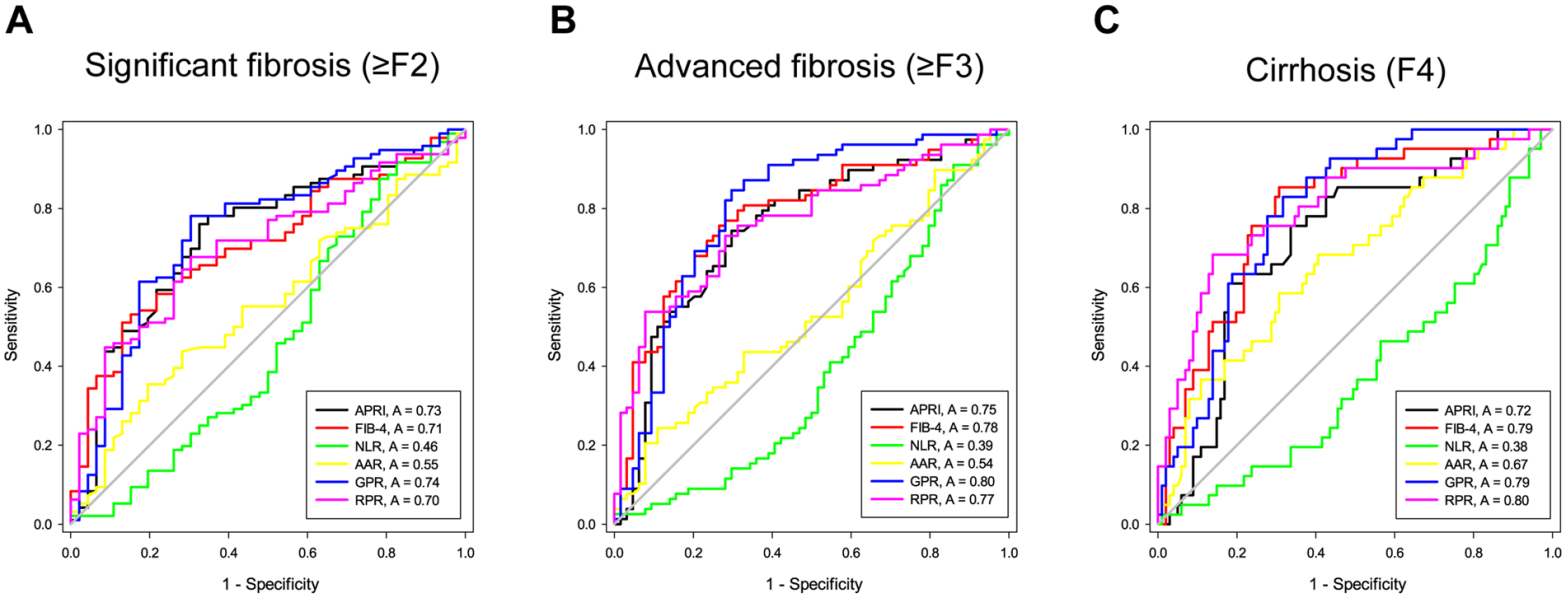
Receiver operating characteristic (ROC) curves of APRI, FIB-4, NLR, AAR, RPR and GPR in the prediction of significant liver fibrosis (**A**), advanced liver fibrosis (**B**) and liver cirrhosis (**C**) in HBeAg positive CHB.

**Figure 4:**
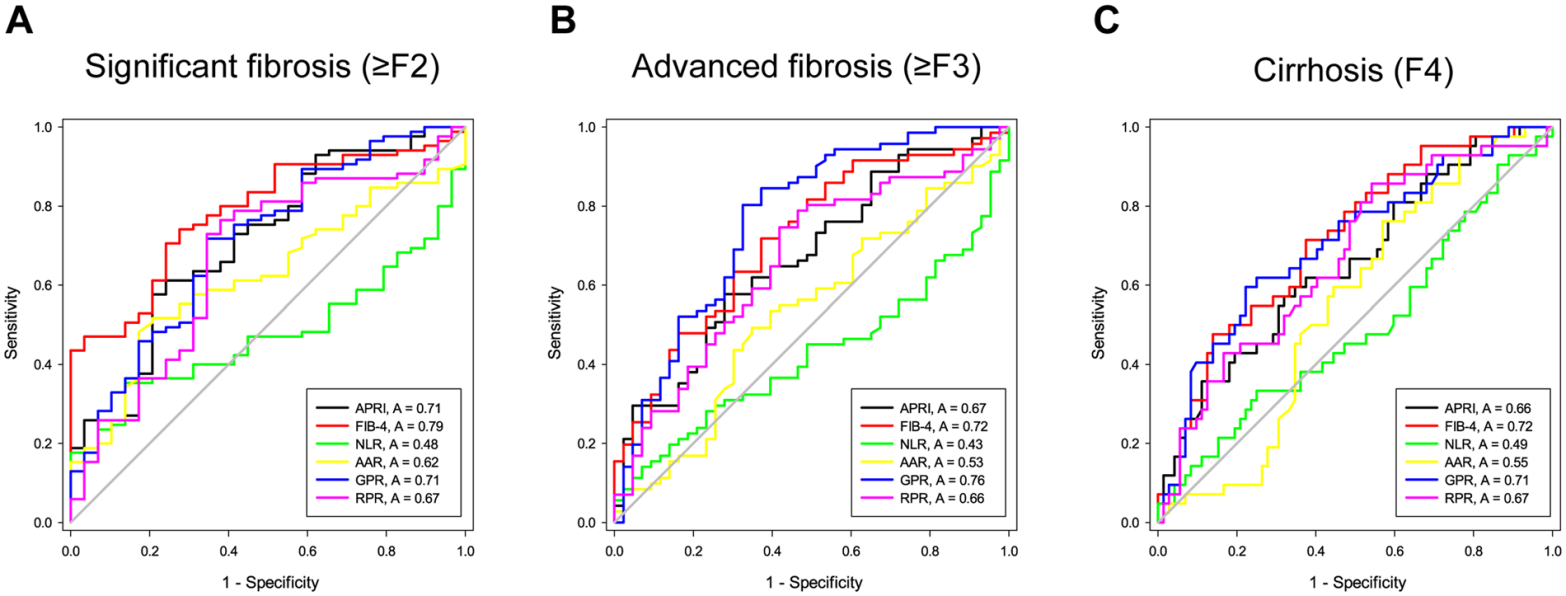
Receiver operating characteristic (ROC) curves of APRI, FIB-4, NLR, AAR, RPR and GPR in the prediction of significant liver fibrosis (**A**), advanced liver fibrosis (**B**) and liver cirrhosis (**C**) in HBeAg negative CHB.

The ROC curves of six models for predicting significant liver fibrosis, advanced liver fibrosis and liver cirrhosis in HBeAg positive CHB and HBeAg negative CHB are shown in Figs 3 and 4, respectively. We compared the diagnostic accuracy of the non-invasive indexes in both HBeAg positive CHB (Table 3) and HBeAg negative CHB (Table 4). In general, the diagnostic performance of GPR was not significantly different with APRI, FIB-4 and RPR in identifying significant fibrosis, advanced fibrosis and cirrhosis, but it was significantly superior to the AAR and NLR.

**Table 3:** Diagnostic accuracy of different indexes for the prediction of liver fibrosis in HBeAg positive CHB.

	Optimized cutoff	Sensitivity (%)	Specificity (%)	AUROC (95% CI)	LR+	LR−	P value	P value of ROC contrast test*
**F0-F1 vs**. **F2-F4**								
APRI	0.561	76.04	67.39	0.730 (0.641, 0.820)	2.332	0.356	<0.001	0.845
	0.5*	79.17	58.70	—	1.917	0.355	<0.001	—
	1.5*	32.29	91.30	—	3.714	0.742	0.002	—
FIB-4	1.594	51.04	86.96	0.706 (0.619, 0.793)	3.914	0.563	<0.001	0.421
	1.45*	57.29	78.26	—	2.635	0.546	<0.001	—
	3.25*	19.79	95.65	—	4.552	0.839	0.015	—
NLR	1.147	87.50	21.74	0.461 (0.351, 0.570)	1.118	0.575	0.447	<0.001
AAR	0.858	35.42	80.43	0.554 (0.455, 0.652)	1.810	0.803	0.301	0.020
RPR	0.075	67.71	69.57	0.702 (0.614, 0.790)	2.225	0.464	<0.001	0.436
GPR	0.350	78.13	69.57	0.737 (0.646, 0.828)	2.568	0.314	<0.001	—
**F0-F2 vs**. **F3-F4**								
APRI	0.672	74.36	70.31	0.751 (0.668, 0.834)	2.505	0.365	<0.001	0.152
FIB-4	1.095	75.64	73.44	0.781 (0.703, 0.859)	2.848	0.332	<0.001	0.568
NLR	1.081	91.03	14.06	0.394 (0.298, 0.490)	1.059	0.638	0.030	<0.001
AAR	1.023	24.36	89.06	0.541 (0.446, 0.637)	2.227	0.849	0.401	<0.001
RPR	0.093	53.85	92.19	0.765 (0.6870, 0.843)	6.895	0.501	<0.001	0.405
GPR	0.413	84.62	70.31	0.801 (0.724, 0.878)	2.850	0.219	<0.001	—
**F0-F3 vs**. **F4**								
APRI	1.087	60.98	82.18	0.718 (0.627, 0.810)	3.422	0.475	<0.001	0.042
	1.0*	60.98	79.21	—	2.932	0.493	<0.001	—
	2.0*	26.83	84.16	—	1.694	0.869	0.131	—
FIB-4	1.349	85.37	69.31	0.792 (0.711, 0.873)	2.782	0.211	<0.001	0.984
NLR	0.767	100.00	2.97	0.381 (0.279, 0.483)	1.031	<0.001	0.026	<0.001
AAR	0.802	58.54	69.31	0.668 (0.569, 0.767)	1.908	0.598	0.002	0.070
RPR	0.096	68.29	86.14	0.799 (0.713, 0.886)	4.927	0.368	<0.001	0.879
GPR	0.645	82.93	68.32	0.793 (0.719, 0.867)	2.618	0.250	<0.001	—

*Compared with GPR.

AUROC, area under the receiver operating characteristic curve; CI, confidence interval; LR−, negative likelihood ratio; LR+, positive likelihood ratio; AAR, aspartate aminotransferase/alanine aminotransferase ratio; APRI, aspartate aminotransferase-to-platelet ratio index; FIB-4, fibrosis index based on the four factors; GPR, gamma-glutamyl transpeptidase to platelet ratio; NLR, neutrophil-to-lymphocyte ratio; RPR, red cell distribution width-to-platelet ratio. *Threshold recommended by the WHO^12^.

**Table 4:** Diagnostic accuracy of different indexes for the prediction of liver fibrosis in HBeAg negative CHB.

	Optimized cutoff	Sensitivity (%)	Specificity (%)	AUROC (95% CI)	LR+	LR−	P value	P value of ROC contrast test*
**F0-F1 vs**. **F2-F4**								
APRI	0.553	61.18	75.86	0.709 (0.599, 0.820)	2.534	0.512	0.001	0.994
	0.5*	69.41	58.62	—	1.677	0.522	0.007	—
	1.5*	17.65	100.00	—	—	0.824	0.035	—
FIB-4	1.328	74.12	72.41	0.785 (0.698, 0.873)	2.687	0.357	<0.001	0.240
	1.45*	65.88	75.86	—	2.729	0.450	<0.001	—
	3.25*	22.35	100.00	—	—	0.776	0.012	—
NLR	2.321	35.29	86.21	0.477 (0.372, 0.582)	2.559	0.751	0.713	0.002
AAR	0.916	51.76	79.31	0.621 (0.512, 0.729)	2.502	0.608	0.053	0.336
RPR	0.072	76.47	62.07	0.667 (0.549, 0.784)	2.016	0.379	0.008	0.523
GPR	0.413	71.76	65.52	0.709 (0.598, 0.820)	2.081	0.431	0.001	—
**F0-F2 vs**. **F3-F4**								
APRI	0.657	57.75	72.09	0.671 (0.570, 0.772)	2.505	0.365	0.002	0.053
FIB-4	1.411	71.83	62.79	0.717 (0.620, 0.813)	2.848	0.332	<0.001	0.434
NLR	3.228	14.08	93.02	0.426 (0.321, 0.530)	1.059	0.638	0.185	<0.001
AAR	0.916	49.30	65.12	0.533 (0.423, 0.644)	2.227	0.849	0.551	0.008
RPR	0.075	74.65	58.14	0.658 (0.555, 0.762)	6.895	0.501	0.005	0.065
GPR	0.413	80.28	67.44	0.764 (0.669, 0.859)	2.850	0.219	<0.001	—
**F0-F3 vs**. **F4**								
APRI	0.724	57.14	68.06	0.658 (0.554, 0.762)	1.789	0.630	0.005	0.314
	1.0*	42.86	77.78	—	1.929	0.735	0.020	—
	2.0*	11.90	98.61	—	8.571	0.893	0.047	—
FIB-4	1.626	71.43	62.50	0.716 (0.621, 0.812)	1.905	0.457	<0.001	0.858
NLR	2.370	33.33	75.00	0.494 (0.380, 0.607)	1.333	0.889	0.911	0.011
AAR	0.700	76.19	43.06	0.549 (0.443, 0.655)	1.338	0.553	0.381	0.053
RPR	0.072	85.71	45.83	0.669 (0.566, 0.771)	1.582	0.312	0.003	0.487
GPR	0.693	59.52	77.78	0.706 (0.606, 0.807)	2.679	0.520	<0.001	—

*Compared with GPR.

AUROC, area under the receiver operating characteristic curve; CI, confidence interval; LR−, negative likelihood ratio; LR+, positive likelihood ratio; AAR, aspartate aminotransferase/alanine aminotransferase ratio; APRI, aspartate aminotransferase-to-platelet ratio index; FIB-4, fibrosis index based on the four factors; GPR, gamma-glutamyl transpeptidase to platelet ratio; NLR, neutrophil-to-lymphocyte ratio; RPR, red cell distribution width-to-platelet ratio. *Threshold recommended by the WHO^12^.

For predicting significant fibrosis, the AUROC of GPR (0.737, 95% CI 0.646 to 0.828) was significantly higher than of NLR (0.461, 95% CI 0.351 to 0.570, P < 0.001), AAR (0.554, 95% CI 0.455 to 0.652, P = 0.020), but was comparable with APRI (0.730, 95% CI 0.641 to 0.820, P = 0.845), FIB-4 (0.706, 95% CI 0.619 to 0.793, P = 0.421) and RPR (0.702, 95% CI 0.614 to 0.790, P = 0.436) in HBeAg positive CHB. In HBeAg negative CHB, the AUROC of GPR (0.709, 95% CI 0.598 to 0.820) was also significantly higher than of NLR (0.477, 95% CI 0.372 to 0.582, P = 0.002), but was similar to APRI (0.709, 95% CI 0.599 to 0.820, P = 0.994), FIB-4 (0.785, 95% CI 0.698 to 0.873, P = 0.240), AAR (0.621, 95% CI 0.512 to 0.729, P = 0.336) and RPR (0.667, 95% CI 0.549 to 0.784, P = 0.523).

For predicting advanced fibrosis, AUROC of GPR was significantly higher than that of NLR and AAR, but was comparable with that of APRI, FIB-4 and RPR in both HBeAg positive CHB and HBeAg negative CHB.

For predicting cirrhosis, AUROC of GPR (0.793, 95% CI 0.719 to 0.867) was significantly higher than that of NLR (0.381, 95% CI 0.279 to 0.483, P < 0.001) and slightly higher than that of APRI (0.718, 95% CI 0.627 to 0.810, P = 0.042), but was similar with FIB-4 (0.792, 95% CI 0.711 to 0.873, P = 0.984) and AAR (0.668, 95% CI 0.569 to 0.767, P = 0.070) and RPR (0.799, 95% CI 0.713 to 0.886, P = 0.879) in HBeAg positive CHB. In HBeAg negative CHB, the AUROC of GPR (0.706, 95% CI 0.606 to 0.807) was also higher than that of NLR (0.494, 95% CI 0.380 to 0.607, P = 0.011) and was comparable with APRI (0.658, 95% CI 0.554 to 0.762, P = 0.314), FIB-4 (0.716, 95% CI 0.621 to 0.812, P = 0.858), AAR (0.549, 95% CI 0.443 to 0.655, P = 0.053) and RPR (0.669, 95% CI 0.566 to 0.771, P = 0.487).

For both HBeAg positive CHB and HBeAg negative CHB, the APRI and FIB-4 could detect significant fibrosis and cirrhosis with only moderate sensitivities and specificities according to the lower thresholds recommended by the WHO in both HBeAg positive CHB and HBeAg negative CHB. The higher threshold yielded a sensitivity of merely 10–20%, although the specificity was relatively high at nearly 80–100%. GPR was not significantly better than the WHO recommended non-invasive tests for detection of fibrosis in both HBeAg positive CHB and HBeAg negative CHB.

## Discussion

Early diagnosis and accuracy assessment of liver fibrosis or cirrhosis may play an important role not only in the control of disease progression, but also in therapeutic judgment for chronic HBV infection^3^. LB is considered as the gold standard for diagnosing liver fibrosis for CHB patients. However, sampling errors, poor reproducibility and invasiveness have limited its use^3^. Many investigators have attempted to propose noninvasive methods to assess liver fibrosis. The perfect noninvasive method should be simple, inexpensive, reliable and accurate in staging liver fibrosis^13^. Several non-invasive methods such as APRI, FIB-4, AAR, NLR and RPR using laboratory tests to predict liver fibrosis have been proposed over the past decade. The application of these indexes may reduce the need for liver biopsy in CHB patients especially in resource-limited settings.

APRI and FIB-4 are the two noninvasive methods for diagnosis liver fibrosis receiving the most attention. Teshale *et al*. determined the predictive ability of APRI and FIB-4 in a large cohort of CHB patients and they found that APRI and FIB-4 distinguished F2–F4 from F0-F1 with good sensitivity and specificity^19^. APRI and FIB-4 were also reported to have a high AUROC to detect significant fibrosis and cirrhosis in patients with CHB in East Africa and Asia^20, 21^. Given that the APRI and FIB-4 are readily available, they have been used in clinical practice. The WHO CHB guidelines have also recommend APRI to determine fibrosis stage in resource-limited countries^12^. However, recently, a meta-analysis suggested that APRI and FIB-4 could identify liver fibrosis with a only moderate sensitivity and accuracy in CHB patients, and were not an ideal replacement for liver biopsy^13^. In the present study, we also analyzed the diagnostic performances of APRI and FIB-4 according to the thresholds recommended by the WHO. For both HBeAg positive CHB and HBeAg negative CHB, by the lower thresholds, the APRI and FIB-4 could detect significant fibrosis and cirrhosis with only moderate sensitivities and specificities. Although, the higher thresholds yielded a specificity of 80–100%, the sensitivity was only 10–20% both to detect significant fibrosis and cirrhosis. Our results were consistent with others^12^. Recently, another study also revealed that the sensitivity for APRI was low and only 10% of patients with cirrhosis were detected using APRI at the WHO recommended threshold in patients with CHB in East Africa^20^.

GPR is a novel index to estimate liver fibrosis in patients with CHB in West Africa cohorts^15^. In the study by Lemoine *et al*., GPR was showed to be more accurate than classical biomarkers APRI and FIB-4^15^. Furthermore, GPR are reported to predict significant liver fibrosis and cirrhosis well in a large cohort of HBV mono-infected Gambian patients using locally validated FibroScan measures as a reference^22^. However, it did show controversial advantage in a Brazilian cohort and other Chinese cohorts^16, 17, 23, 24^. We performed a validation analysis for GPR in the present study. In our study, although the AUROC of GPR was slightly higher than APRI in predicting advanced fibrosis and cirrhosis in CHB patients, it was not superior to the FIB-4 in identifying significant fibrosis, advanced fibrosis and cirrhosis in CHB patients in China.

To determine why the GPR, which shows application prospect in West Africa, is not useful in other cohorts, we compared the baseline characteristics of the study population of these studies^15–17^. We found that one important factor was that the HBeAg states of patients were quite different in these studies^15–17^. In the study by Lemoine *et al*., most of patients are HBeAg seronegative in the Gambia cohort (96%) and Senegal cohort (83%)^15^. But, the HBeAg seropositive is 57.5% and 53% in the cohorts reported by Li *et al*. and Schiavon et al., respectively^16, 17^. Thus, we compared the diagnostic accuracy of GPR with other non-invasive indexes according to the HBeAg states. For HBeAg positive CHB, the diagnostic performances of GPR was superior to APRI in identifying cirrhosis but was comparable with FIB-4 in identifying significant fibrosis, advanced fibrosis and cirrhosis. For HBeAg negative CHB, the GPR does not show any advantages than APRI and FIB-4 to predict significant fibrosis, advanced fibrosis and cirrhosis. Thus, the HBeAg states are not responsible for the discrepancies. We consider these discrepancies might be related to variations in inclusion and exclusion criteria for patients, laboratory methods, HBV genotypes, different histological scoring systems and even demographic characteristics.

AAR, NLR and RPR are also proposed to predict the degrees of liver fibrosis recent years. However, in our study, the relationship between degrees of liver fibrosis and the AAR scores was not significant, indicating that AAR is not a good method for the estimation of fibrosis stage in patients with CHB. Our results are consistent with others^25, 26^. The NLR could predict the advanced fibrosis and cirrhosis in HBeAg positive CHB. However, the specificity was very low (14.06% and 2.97%, respectively) and it did not predict the degrees of liver fibrosis in HBeAg negative CHB in our study. GPR was significantly superior to AAR and NLR in predicting the degrees of liver fibrosis. The RPR was reported to predict significant fibrosis and cirrhosis in CHB patients with relatively high accuracy by several studies^18, 27, 28^. In our study, we also found that the RPR could predict significant fibrosis, advanced fibrosis and cirrhosis with a relative high accuracy in both HBeAg positive CHB and HBeAg negative CHB. Few study compared the diagnostic accuracy in predicting the degrees of liver fibrosis between RPR and GPR. GPR did not show any advantage to RPR in predicting significant fibrosis, advanced fibrosis and cirrhosis in both HBeAg positive CHB and HBeAg negative CHB in our study.

There are several limitations to this study that warrant consideration. Firstly, the study is a retrospective study and the patients were enrolled retrospectively. Secondly, the HBV genotypes of the patients were not assessed. The determination of the HBV genotype is not a routine clinical test. Thirdly, the GPR was not dynamically observed. Whether GPR may be superior to other NITs in evaluating regression of fibrosis after long-term antiviral suppression of HBV or predicting liver-related end-points, such as hepatic decompensation or HCC deserves further investigation. Finally, this study involved a single center which may be subjected to selection bias.

In summary, although GPR showed an acceptable diagnostic performance for the detection of advanced liver fibrosis in Chinese patients with CHB and represent a routinely available, inexpensive and easily calculated index, it does not show advantages than APRI, FIB-4 and RPR in identifying significant fibrosis, advanced fibrosis and cirrhosis in both HBeAg positive CHB and HBeAg negative CHB. However, it should also be noted that the sample size was relatively small in our study. Thus, the superiority of the predictive performance of GPR should be further confirmed in multi-center, prospectively studies with larger sample sizes. Furthermore, these non-invasive methods (GPR, APRI, FIB-4 and RPR) generally can give moderate estimates in the diagnosis of significant fibrosis and liver cirrhosis. These non-invasive methods should be use with cautious in CHB patients. Serum biomarkers based on blood or serum parameters may be used in combination with other non-invasive tests such as imaging or elastography to improve the diagnostic accuracy of liver fibrosis.

## Patients and Methods

### Patients

We included the patients who underwent LB from the Huai’an No. 4 People’s Hospital between the years 2008–2015 with the initial diagnosis of CHB. CHB was defined as the persistent presence of serum HBV surface antigen (HBsAg) for >6 months. Liver histology of the CHB patients was done before any initiation of antiviral therapy.

Patients with the following conditions were excluded from the study: (i) blood transfusion 3 months prior to admission; (ii) malignant diseases including liver cancer; (iii) hematological diseases; (iv) co-infection with hepatitis C virus, hepatitis D virus, hepatitis E virus or HIV; (v) coexistence of autoimmune hepatitis, alcoholic liver disease, drug hepatitis, Wilson’s disease or syphilis; (vi) liver transplantation and (vii) cardiovascular diseases.

This study was conducted in accordance with the ethics principles of the Declaration of Helsinki and was approved by the Ethics Committee of Huai’an No. 4 People’s Hospital. Written informed consent was obtained from all patients according to standards of the local ethics committees. This study had no influence on the subsequent management of patients.

### Data collection

We retrospectively reviewed the medical records of the included patients. The demographic, clinical and laboratory characteristics including routine blood test, liver enzymes, HBV serological markers and HBV DNA levels were recorded. The degrees of hepatic inflammation and fibrosis of all the patients were collected. The METAVIR scoring system was used as the pathological diagnosis standard of liver fibrosis. The liver fibrosis was classified into the following five stages: F0, no fibrosis; F1, portal fibrosis without septa; F2, portal fibrosis with rare septa; F3, numerous septa without cirrhosis; and F4, cirrhosis^29^. The fibrotic stages were assessed by two experienced pathologists who were fully blinded to any clinical data according to the above-mentioned criteria.

### Non-invasive prediction methods and calculation formulae

The non-invasive prediction methods and calculation formulae were as follows:

AAR = AST (U/L)/ALT (U/L)^30^; GPR = (GGT (U/L)/ULN of GGT)/platelet count (10^9^/L) × 100^15, 16^; APRI = (AST (U/L)/ULN of AST)/platelet count (10^9^/L) × 100^12^; FIB-4 = (age (years) × AST (U/L))/ ((platelet count (10^9^/L) × (ALT (U/L))^1/2^)^12^; NLR = neutrophil count (10^9^/L)/lymphocyte count (10^9^/L)^31, 32^; RPR = RDW (%)/platelet count (10^9^/L)^18, 27, 28^.

### Statistical analyses

The data analysis was performed using SPSS version 22.0 software (SPSS Inc., Chicago, IL, United States) and SigmaPlot version 12.5 (Systat Software Inc., San Jose, CA, United States). Continuous variables were expressed as mean value ± standard deviation for normal distribution data or non-normal distribution continuous data as median (interquartile range (IQR)). Categorical data were reported as percentages. Correlations were evaluated by the Spearman’s rank correlation coefficient analysis. The diagnostic performance of serum model for liver fibrosis was estimated by the receiver operating characteristic (ROC) curve and the area under the ROC curve (AUROC). Differences between the AUROCs were tested using the z-test. The cut-off values were determined by the Youden index which was the optimal combination of sensitivity and specificity. The sensitivity, specificity, positive likelihood ratio and negative likelihood ratio were also calculated based on established thresholds proposed by WHO CHB guidelines: 0.5 and 1.5 to distinguish F0-1 and F2-4,1.0 and 2.0 to differentiate F0-3 and F4 for APRI, 1.45 and 3.25 to distinguish F0-2 and F3-4 for FIB-4^12^. All P-values were 2-sided and any value of P < 0.05 was considered statistically significant.

## Electronic supplementary material

Supplementary material

Supplementary Dataset 1

## References

[1] Liaw Y-F, Chu C-M (2009). Hepatitis B virus infection. Lancet..

[2] Trépo C, Chan HLY, Lok A (2014). Hepatitis B virus infection. Lancet..

[3] Shiha G (2016). Asian-Pacific Association for the Study of the Liver (APASL) consensus guidelines on invasive and non-invasive assessment of hepatic fibrosis: a 2016 update. Hepatol. Int..

[4] Intraobserver and interobserver variations in liver biopsy interpretation in patients with chronic hepatitis C. The French METAVIR Cooperative Study Group. *Hepatology* **20**, 15–20, doi:10.1002/hep.1840200104 (1994). 8020885

[5] Cadranel JF, Rufat P, Degos F (2000). Practices of liver biopsy in France: results of a prospective nationwide survey. For the Group of Epidemiology of the French Association for the Study of the Liver (AFEF). Hepatology.

[6] Regev A (2002). Sampling error and intraobserver variation in liver biopsy in patients with chronic HCV infection. Am. J. Gastroenterol..

[7] Kim JH, Kim MN, Han K-H, Kim SU (2015). Clinical application of transient elastography in patients with chronic viral hepatitis receiving antiviral treatment. Liver Int..

[8] Seo YS (2015). Accuracy of transient elastography in assessing liver fibrosis in chronic viral hepatitis: A multicentre, retrospective study. Liver Int..

[9] Millonig G (2008). Extrahepatic cholestasis increases liver stiffness (FibroScan) irrespective of fibrosis. Hepatology.

[10] Kim SU (2009). Liver stiffness measurement using FibroScan is influenced by serum total bilirubin in acute hepatitis. Liver Int..

[11] Fung J (2011). Mild-to-moderate elevation of alanine aminotransferase increases liver stiffness measurement by transient elastography in patients with chronic hepatitis B. Am. J. Gastroenterol..

[12] World Health Organization. Guidelines for the Prevention, Care and Treatment of Persons with Chronic Hepatitis B Infection (2015). 26225396

[13] Xiao G, Yang J, Yan L (2015). Comparison of diagnostic accuracy of aspartate aminotransferase to platelet ratio index and fibrosis-4 index for detecting liver fibrosis in adult patients with chronic hepatitis B virus infection: a systemic review and meta-analysis. Hepatology.

[14] Kim WR (2016). Evaluation of APRI and FIB-4 scoring systems for non-invasive assessment of hepatic fibrosis in chronic hepatitis B patients. J. Hepatol..

[15] Lemoine M (2016). The gamma-glutamyl transpeptidase to platelet ratio (GPR) predicts significant liver fibrosis and cirrhosis in patients with chronic HBV infection in West Africa. Gut.

[16] Li Q (2016). The Gamma-Glutamyl-Transpeptidase to Platelet Ratio Does not Show Advantages than APRI and Fib-4 in Diagnosing Significant Fibrosis and Cirrhosis in Patients With Chronic Hepatitis B: A Retrospective Cohort Study in China. Medicine.

[17] Schiavon, L. L., Narciso-Schiavon, J. L., Ferraz, M. L. G., Silva, A. E. B. & Carvalho-Filho, R. J. The γ-glutamyl transpeptidase to platelet ratio (GPR) in HBV patients: just adding up? *Gut*. doi:10.1136/gutjnl-2016-312658 (2016). 10.1136/gutjnl-2016-312658 27534672

[18] Chen B, Ye B, Zhang J, Ying L, Chen Y (2013). RDW to platelet ratio: a novel noninvasive index for predicting hepatic fibrosis and cirrhosis in chronic hepatitis B. PloS One.

[19] Teshale E (2014). APRI and FIB-4 are good predictors of the stage of liver fibrosis in chronic hepatitis B: the Chronic Hepatitis Cohort Study (CHeCS). J. Viral Hepat..

[20] Desalegn H, Aberra H, Berhe N, Gundersen SG, Johannessen A (2017). Are non-invasive fibrosis markers for chronic hepatitis B reliable in sub-Saharan Africa?. Liver Int..

[21] Shin WG (2008). Aspartate aminotransferase to platelet ratio index (APRI) can predict liver fibrosis in chronic hepatitis B. Dig. Liver Dis..

[22] Lemoine, M., Thursz, M., Mallet, V. & Shimakawa, Y. Diagnostic accuracy of the gamma-glutamyl transpeptidase to platelet ratio (GPR) using transient elastography as a reference. *Gut* doi:10.1136/gutjnl-2016-311554 (2016). 10.1136/gutjnl-2016-311554 26921348

[23] Zhu, M.-Y. *et al*. A novel noninvasive algorithm for the assessment of liver fibrosis in patients with chronic hepatitis B virus infection. *J*. *Viral Hepat*., doi:10.1111/jvh.12682 (2017). 10.1111/jvh.12682 28130852

[24] Cai, Y.-J. *et al*. A diagnostic algorithm for assessment of liver fibrosis by liver stiffness measurement in patients with chronic hepatitis B. *J*. *Viral Hepat*., doi:10.1111/jvh.12715 (2017). 10.1111/jvh.12715 28419755

[25] Eminler AT (2015). AST/ALT ratio is not useful in predicting the degree of fibrosis in chronic viral hepatitis patients. Eur. J. Gastroenterol. Hepatol..

[26] Vardar R (2009). Is there any non-invasive marker replace the needle liver biopsy predictive for liver fibrosis, in patients with chronic hepatitis?. Hepatogastroenterology..

[27] Xu W-S (2015). Red blood cell distribution width levels correlate with liver fibrosis and inflammation: a noninvasive serum marker panel to predict the severity of fibrosis and inflammation in patients with hepatitis B. Medicine.

[28] Taefi A, Huang C-C, Kolli K, Ebrahimi S, Patel M (2015). Red cell distribution width to platelet ratio, a useful indicator of liver fibrosis in chronic hepatitis patients. Hepatol. Int..

[29] Bedossa P, Poynard T (1996). An algorithm for the grading of activity in chronic hepatitis C. The METAVIR Cooperative Study Group. Hepatology.

[30] Giannini E (2003). Validity and clinical utility of the aspartate aminotransferase-alanine aminotransferase ratio in assessing disease severity and prognosis in patients with hepatitis C virus-related chronic liver disease. Arch. Intern. Med..

[31] Biyik M (2013). Blood neutrophil-to-lymphocyte ratio independently predicts survival in patients with liver cirrhosis. Eur. J. Gastroenterol. Hepatol..

[32] Alkhouri N (2012). Neutrophil to lymphocyte ratio: a new marker for predicting steatohepatitis and fibrosis in patients with nonalcoholic fatty liver disease. Liver Int..

