# Supplementary material for: Gamma-glutamyl-transpeptidase to platelet ratio is not superior to APRI,FIB-4 and RPR for diagnosing liver fibrosis in CHB patients in China
Source: Sci Rep. 2017 Aug 17;7:8543. doi: 10.1038/s41598-017-09234-w (PMC5561053; doi:10.1038/s41598-017-09234-w)
Supplement: Supplementary file 1 — Supplementary material [file 41598_2017_9234_MOESM1_ESM.pdf]

Gamma-glutamyl-transpeptidase to platelet ratio is not superior to APRI, FIB-4 and RPR for diagnosing liver fibrosis in CHB patients in China

Rui Huang, Guiyang Wang, Chen Tian, Yong Liu, Bei Jia, Jian Wang, Yue Yang, Yang Li, Zhenhua Sun, Xiaomin Yan, Juan Xia, Yali Xiong, Peixin Song, Zhaoping Zhang, Weimao Ding, Chao Wu

Table of Contents **Page**

Supplementary Table S1. Comparisons of baseline characteristics between HBeAg positive CHB and HBeAg negative CHB. **2**

Supplementary Fig. S1. Flow chart describing the selection of the study population. **3**

Supplementary Fig. S2. Correlations between liver fibrosis stages and different NITs. **4**

Supplementary Table S1. Comparisons of baseline characteristics between HBeAg positive CHB and HBeAg negative CHB

Characteristic	HBeAg positive CHB (n=142)	HBeAg negative CHB (n=114)	P values
Median age (years) (IQR)	34.0 (27.0, 42.0)	43.5 (36.0, 48.0)	< 0.001
Male (%)	118 (83.1)	86 (79.7)	0.130
Median ALT (IU/L) (IQR)	54.5 (34.8, 102.0)	36.5 (23.8, 60.3)	< 0.001
Median AST (IU/L) (IQR)	39.0 (27.8, 68.0)	32.0 (24.0, 44.5)	< 0.001
Median GGT (IU/L) (IQR)	41.0 (22.8, 96.5)	35.0 (22.8, 70.3)	0.321
Median Neutrophils ($\times10^9$/L) (IQR)	2.9 (2.1, 3.5)	2.9 (2.3, 3.6)	0.794
Median Lymphocytes ($\times10^9$/L) (IQR)	1.8 (1.4, 2.1)	1.6 (1.3, 2.0)	0.012
Median Hb (g/L) (IQR)	146.0 (136.0,155.3)	142.0 (136.0, 153.0)	0.088
Median PLT ($\times10^9$/L) (IQR)	151.0 (119.5, 202.3)	146.0 (97.8, 186.3)	0.166
Median RDW (%) (IQR)	12.0 (11.5, 12.5)	12.0 (11.5, 12.7)	0.448
Tbil (μmol/L) (IQR)	14.7 (11.7, 21.5)	15.9 (11.4, 21.2)	0.856
Median size of liver biopsy (cm) (IQR)	1.4 (1.0, 1.6)	1.3 (1.0, 1.5)	0.202
Fibrosis stages			0.508
F0 (%)	23 (16.2)	11 (9.6)	
F1 (%)	23 (16.2)	18 (15.8)	
F2 (%)	18 (12.7)	14 (12.3)	
F3 (%)	37 (26.1)	29 (25.4)	
F4 (%)	41 (28.9)	42 (36.8)	
Median HBV DNA level (log10 copies/mL) (IQR)	6.6 (5.4, 7.4)	3.3 (3.0, 4.8)	< 0.001

ALT, alanine aminotransferase; AST, aspartate aminotransferase; CHB, chronic hepatitis B; GGT, gamma-glutamyl transferase; Hb, hemoglobin; IQR, interquartile range; PLT, platelets; RDW, red cell distribution width; Tbil, total bilirubin.

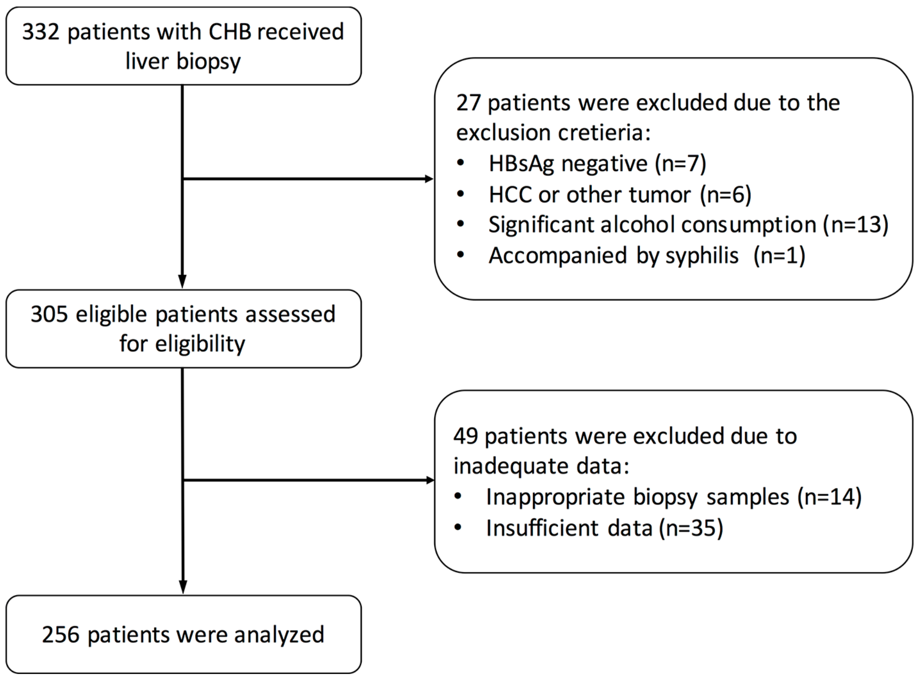

Supplementary Fig. S1. Flow chart describing the selection of the study population.

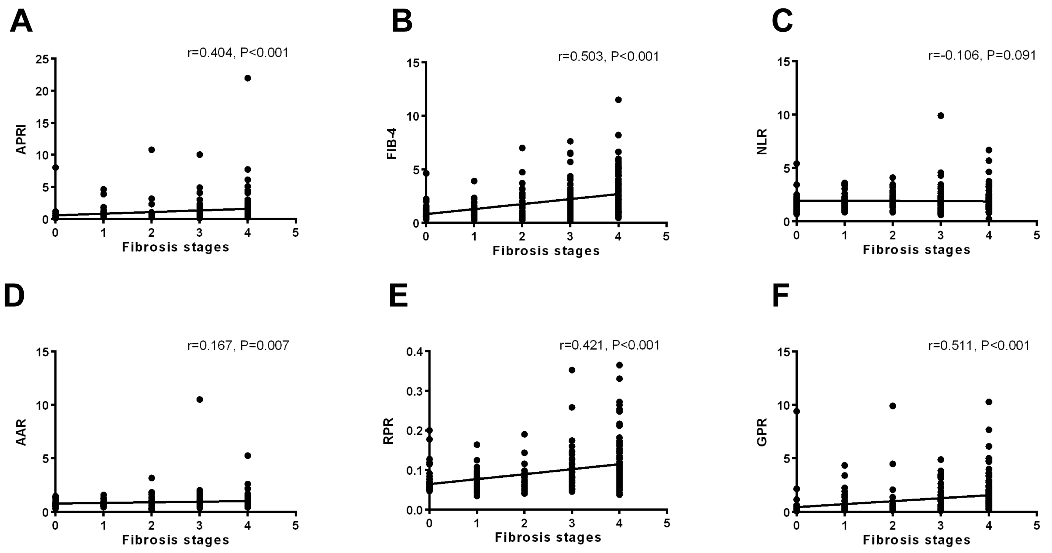

Supplementary Fig. S2. Correlations between liver fibrosis stages and different NITs.
